# Novel bacteriocins from lactic acid bacteria (LAB): various structures and applications

**DOI:** 10.1186/1475-2859-13-S1-S3

**Published:** 2014-08-29

**Authors:** Rodney H Perez, Takeshi Zendo, Kenji Sonomoto

**Affiliations:** 1 Laboratory of Microbial Technology, Division of Systems Bioengineering, Department of Bioscience and Biotechnology, Faculty of Agriculture, Graduate School, Kyushu University, 6-10-1 Hakozaki, Higashi-ku, Fukuoka 812-8581, Japan; 2 Laboratory of Functional Food Design, Department of Functional Metabolic Design, Bio-Architecture Center, Kyushu University, 6-10-1 Hakozaki, Higashi-ku, Fukuoka 812-8581, Japan

**Keywords:** bacteriocin, lactic acid bacteria, screening of novel bacteriocin, bacteriocin application

## Abstract

Bacteriocins are heat-stable ribosomally synthesized antimicrobial peptides produced by various bacteria, including food-grade lactic acid bacteria (LAB). These antimicrobial peptides have huge potential as both food preservatives, and as next-generation antibiotics targeting the multiple-drug resistant pathogens. The increasing number of reports of new bacteriocins with unique properties indicates that there is still a lot to learn about this family of peptide antibiotics. In this review, we highlight our system of fast tracking the discovery of novel bacteriocins, belonging to different classes, and isolated from various sources. This system employs molecular mass analysis of supernatant from the candidate strain, coupled with a statistical analysis of their antimicrobial spectra that can even discriminate novel variants of known bacteriocins. This review also discusses current updates regarding the structural characterization, mode of antimicrobial action, and biosynthetic mechanisms of various novel bacteriocins. Future perspectives and potential applications of these novel bacteriocins are also discussed.

## Introduction

Bacteriocins comprise a huge family of ribosomally synthesized peptides that have antibacterial activity towards closely related strains [1,2], although there are an increasing number of bacteriocins reported to have broad range antimicrobial activity [3,4]. In the past decade, interest in bacteriocin research, especially from lactic acid bacteria (LAB), has gained great momentum due to its potential as both a natural food preservative and as therapeutic antibiotics [2,5-8]. Bacteriocins have a number of positive attributes that have made them especially attractive for various applications. LAB bacteriocins are inherently tolerant to high thermal stress and are known for their activity over a wide pH range. These antimicrobial peptides are also colourless, odourless, and tasteless, which further enhance their potential usefulness. Despite the long history of bacteriocin use, there have been no reports on the development of resistant bacteria. One possible reason is that bacteriocins have a fast acting mechanism, which forms pores in the target membrane of bacteria, even at extremely low concentrations. They are also easily degraded by proteolytic enzymes due to their proteinaceous nature. Therefore, bacteriocin fragments do not live long in the human body or in the environment, which minimizes the opportunity of target strains to interact with the degraded antibiotic fragments: this is the common starting point in the development of antibiotic resistance. Perhaps, the most significant advantage of bacteriocins over conventional antibiotics is their primary metabolite nature since they have relatively simple biosynthetic mechanisms compared with conventional antibiotics, which are secondary metabolites. This fact makes them easily amenable through bioengineering to increase either their activity or specificity towards target microorganisms. The main differences between bacteriocins and conventional antibiotics are summarized in Table 1. For food applications, LAB bacteriocins have inherent advantage over conventional antibiotics since, unlike the later, LAB bacteriocins are generally considered food-grade due to the typical association of LAB to food fermentation that dates back to ancient times. In fact, the U.S. Food and Drug Administration (FDA) classified LAB and its by-products as Generally Regarded as Safe (GRAS) as a human food ingredient [9]. Whereas for clinical applications, bacteriocins have been presented as a viable alternative to antibiotics due to the high specificity of some bacteriocins against clinical pathogens, including multi-antibiotic resistant (MDR) strains [7].

**Table 1:** Main differences between (LAB) bacteriocins and conventional antibiotics.

Characteristic	Bacteriocins	Antibiotics
Application	Food/Clinical	Clinical
Synthesis	Ribosomal	Secondary metabolite
Bioactivity spectra	Mostly narrow	Mostly broad
Intensity of bioactivity	Active at nano-to-micro molar range	Active at micro-to-milli molar range
Proteolytic enzyme degradability	High	Moderate-to-none
Thermal stability	High	Low
Active pH range	Wide	Narrow
Color/taste/odour	No	Yes
Amenability to bioengineering	Yes	No
Possible mechanism of target cell developing resistance	Adaptation through changes in cell membrane composition	Genetically transferable determinant that inactivates the active compound
Mode of action	Pore formation, inhibition of cell wall biosynthesis	Cell membrane or intercellular targets
Toxicity towards eukaryotic cells	Relatively no	Yes

With the discovery of new bacteriocins that have unique characteristics, it has become apparent that they are a very diverse and heterogeneous group of compounds. Over the years, various schemes have been suggested to classify bacteriocins from Gram-positive bacteria including LAB [10-13]. Cotter *et al*. (2005) suggested a more radical modification of the previous classification scheme [12]. According to this scheme, bacteriocins are grouped into just two categories (Table 2): lantibiotics (class I) and non-lanthionine-containing bacteriocins (class II), as opposed to the four classes of the Klaenhammer classification scheme [10]. The most notable change in this scheme is that it reclassified the class III bacteriocins as bacteriolysins, since they are lytic enzymes rather than peptides. Recently, although this classification scheme was broadly agreed with, Heng *et al*. (2007) suggested a further modification in which circular bacteriocins should be grouped as a different class [13]. Class I bacteriocins or lantibiotics (lanthionine-containing antibiotics) are small peptides (<5 kDa) that possess unusual post-translationally modified residues such as lanthionine or 3-methyllanthionine. These unusual residues form covalent bonds between amino acids, which result in internal "rings" and give lantibiotics their characteristic structural features [12,14]. The most extensively studied bacteriocin, nisin A and its variants are the main representatives of lantibiotics. Class II bacteriocins, or the non-lantibiotics, are the most naturally occurring bacteriocins. They are small (<10 kDa), heat-stable, non-lanthionine-containing peptides, which, unlike lantibiotics do not undergo extensive post-translational modification. This group can be further subdivided into four subclasses: "pediocin-like" bacteriocins (class IIa), two-component bacteriocins (class IIb), circular bacteriocins (class IIc), and unmodified, linear, non-pediocin-like bacteriocins (class IId) [12].

**Table 2:** Bacteriocin classification with representative bacteriocins isolated in our laboratory.

Class		Features	Examples (Reference)
I		Lantibiotics, small (<5 kDa) peptides containing lanthionine and β-methyllanthionine	Nisin Z [77] and Q [31], Enterocin W [78], Nukacin ISK-1 [79]

II		Small (<10 kDa), heat-stable, non-lanthionine-containing peptides	
	
	IIa	Small heat-stable peptides, synthesized in a form of precursor which is processed after two glycine residues, active against *Listeria*, have a consensus sequence of YGNGVXC in the N-terminal	Enterocin NKR-5-3C [35,36], Enterocin A [80], Munditicin [81], Leucocin A [80]
	
	IIb	Two component systems: two different peptides required to form an active poration complex	Lactococcin Q [43], Enterocin NKR-5-3AZ [36], Enterocin X [44]
	
	IIc	N- and C- termini are covalently linked, resulting in a circular structure	Lactocyclicin Q [49], Leucocyclicin Q [50]
	
	IId	Other class II bacteriocins, including *sec*-dependent bacteriocins and leaderless bacteriocins	Lacticin Q [22] and Z [54], Weissellicin Y and M [55], Leucocin Q and N [80]

III ^Ϯ^		Large molecules heat sensitive peptides	

^Ϯ ^suggested to be renamed *Bacteriolysins *.

Class IIa bacteriocins have a distinct conserved sequence (YGNGVXC) in the N-terminal region that is responsible for their high potency against the food pathogen *Listeria monocytogenes *[15,16]. The class IIb bacteriocins are two-peptide bacteriocins that require both peptides to work synergistically to be fully active [17,18]. Class IIc bacteriocins, arguably the most poorly understood of the bacteriocins, are grouped based on their circular structural configuration. The N- and C-termini of class IIc bacteriocins are covalently linked giving the peptide an extremely stable structure [19,20]. On the other hand, class IId bacteriocins comprise the remaining bacteriocins combined as miscellaneous, or as a one-peptide non-pediocin linear group [12]. *Sec-*dependent bacteriocins [21] and leaderless bacteriocins [22] belong to this class.

Despite the huge potential of bacteriocins in a number of applications, nisin remains the only commercially available bacteriocin used for food applications. However, the use of bacteriocins in clinical settings has been limited to animal, rather than human, health [7]. Nevertheless, the approval of nisin for application in food by the Joint Food and Agriculture Organization/World Health Organization Expert Committee on Food Additives, as well as the approval by the US Food and Drug Agency (FDA) for its use in pasteurized, processed cheese spreads, should establish a legal precedent for the use of other bacteriocins as food preservatives. In clinical settings, the increasing number of researchers developing bioengineered bacteriocins with enhanced properties targeting clinical pathogens should fast track the widespread use of bacteriocins. Thus, it is important to discover more novel bacteriocins with unique properties in order to enhance the arsenal of antimicrobials we have for controlling both undesirable bacteria in food and clinical pathogens. In this review we describe an efficient screening system employed in our lab that fast tracks the discovery of novel LAB bacteriocins. We also discuss a few examples of novel bacteriocins that are studied in our lab, describing their structures and characteristics as well as highlighting their exemplary properties that could be deemed useful for their future applications.

## Biology of bacteriocins

Bacteriocin production is widespread among bacteria. It has been suggested that the majority of bacterial species synthesize bacteriocins [6,12]. This is because their biosynthetic machineries are relatively simple and are often associated with transferable elements such as conjugative transposons or plasmids [12]. As highlighted earlier, bacteriocins are ribosomally synthesized peptides. Genes related to bacteriocin biosynthesis are generally clustered, and are encoded on plasmids, chromosome and/or transposons with minimum genetic machinery consisting of structural cognate immunity genes [10]. Bacteriocins are usually synthesized as biologically inactive prepeptides that include an N-terminal leader peptide attached to the C-terminal propeptide [5,10,12]. The leader peptide: (i) serves as a recognition site which directs the prepeptide towards maturation and transport proteins, (ii) protects the producer strain by keeping the bacteriocin in an inactive state while it is inside the producer strain, and (iii) interacts with the propeptide domain to ensure it is in a suitable conformation for enzyme-substrate interaction of the modification machinery [10,23-25].

**Figure 1:**
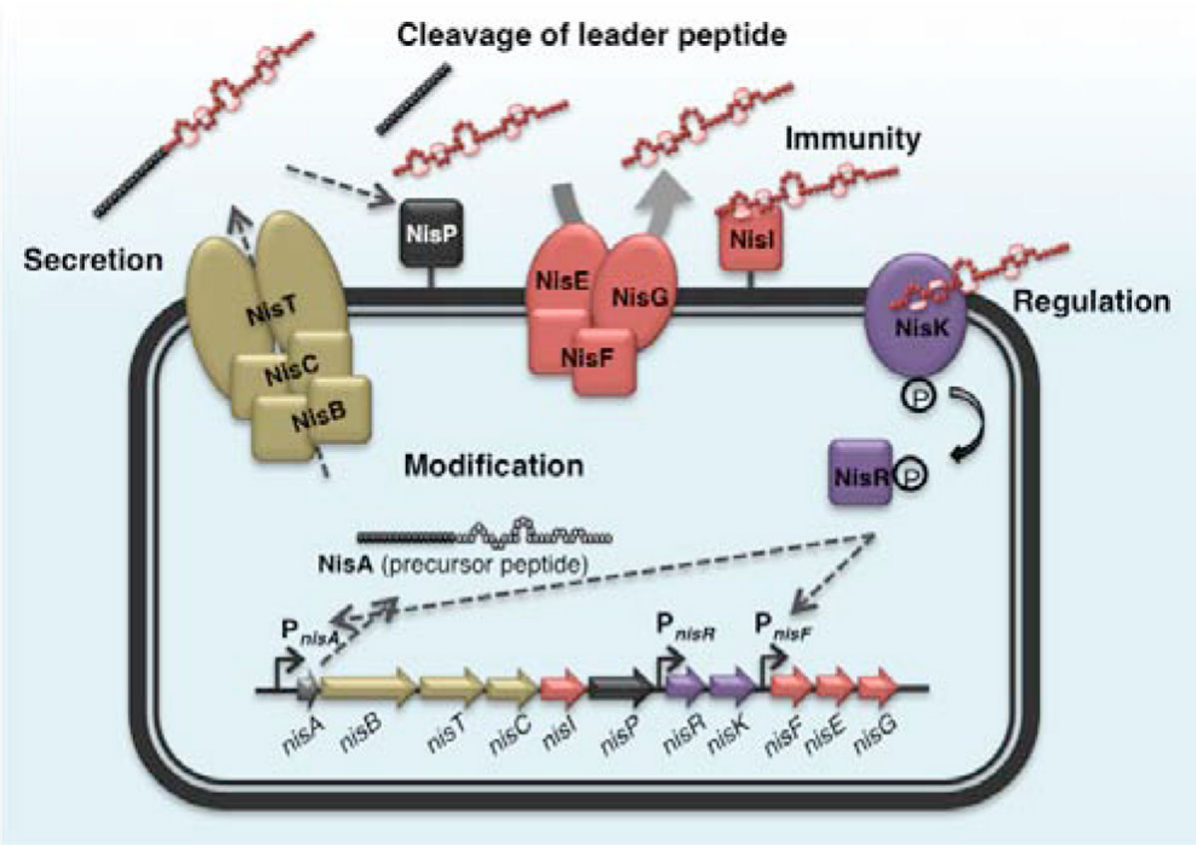
**A schematic diagram of the biosynthesis of nisin A**. Nisin A is ribosomally synthesized as an inactive prepeptide, NisA, consisting of an N-terminal leader sequence attached to a propeptide moiety. Modification enzymes, NisB and NisC, dehydrate and cyclize the propeptide respectively, and subsequently the ABC transporter, NisT, translocates the modified prepeptide into the extracellular space. The protease, NisP, then cleaves off the leader peptide, releasing the mature (active form) nisin A. The lipoprotein NisI that can bind to nisin A, and the multi-protein ABC transporter complex NisFEG, which expels nisin A from the cell, comprise the self-immunity system for nisin A. Two-component regulation system that is responsible for the up-regulation of the nisin gene cluster is composed of a histidine kinase, NisK, and a response regulator, NisR. Nisin A serves as the signal peptide that activates this regulation system.

Lantibiotic biosynthesis begins with translation of the prepeptide, which consists of a leader peptide and a modifiable propeptide moiety. The prepeptide then undergoes modification, following which the modified prepeptide translocates across the cytoplasmic membrane and the leader peptide is cleaved proteolytically by specific enzymes. Genes encoding immunity proteins, as well as proteins involved in the regulation of its production, are normally located in a cluster around the bacteriocin structural gene. As an example, the biosynthetic mechanism of the most extensively studied bacteriocin, nisin A, is illustrated in Figure 1. For a more detailed description, one could refer to the latest comprehensive review on nisin biosynthesis [26].

Like lantibiotics, class II bacteriocins are synthesized as an inactive prepeptide that usually contain a characteristic double-glycine proteolytic processing site [15,16]. However, some class II bacteriocins are synthesized with a typical N-terminal signal sequence of a *sec*-dependent type, and secreted through the general secretory pathway [27]. Unlike lantibiotics, class II bacteriocins do not undergo extensive post-translational modification. Following the translation of the prepeptide, it is processed by specific enzymes to cleave off the leader peptide concomitant with its translocation to the extracellular space through a dedicated ABC-transporter that sometimes require an accessory protein [15,16]. However, there are a growing number of newly reported bacteriocins that lack leader sequences; these are of interest as they are active immediately after translation [21,22,28].

## Fast tracking the screening of novel bacteriocins

Bacteriocins must be obtained in their purified form in order to be studied and characterized. However, it is well known that establishing a purification system for bacteriocins is expensive, time-consuming, and tedious. Moreover, in many cases, these systems often identify previously reported bacteriocins. This has led us to develop a rapid system that can screen for novel bacteriocins from various sources during the early stage of the screening and isolation of bacteriocin-producing strains. This has enabled us to save time, effort, and money in fast tracking the discovery of more novel bacteriocins.

**Figure 2:**
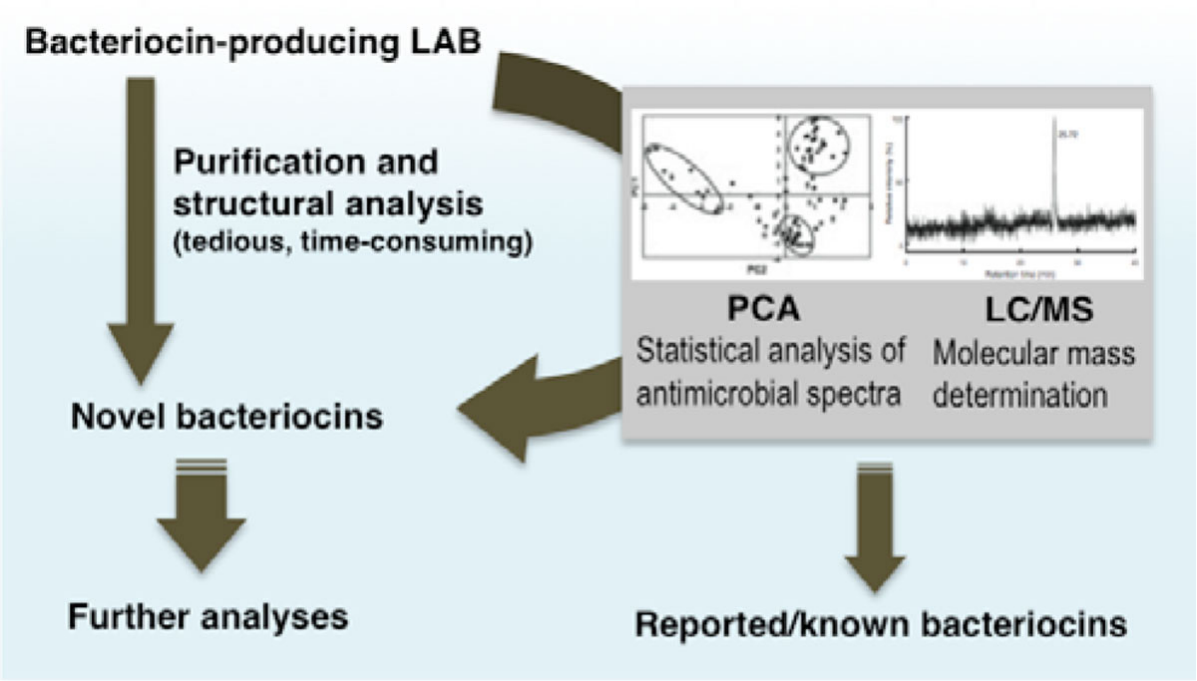
**Rapid screening system for novel LAB bacteriocins**. Bacteriocins from various LAB strains are evaluated for novelty at the early stage of screening. The process of bacteriocin purification and structural analysis are tedious and time-consuming, and in most cases would lead to the isolation of known bacteriocins. Hence, it is necessary to evaluate the novelty as early as possible during the screening process. In this system ESI-LC/MS analyses of the culture supernatant, combined with principal component analysis (PCA) of their activity spectra, are employed. Potential novel bacteriocins are then selected for purification and further analyses.

This system employs molecular mass analyses using electrospray ionization liquid chromatography/mass spectrometry (ESI-LC/MS) in combination with principal component analyses (PCA) of the antimicrobial activity spectrum of each bacteriocin producing LAB strain (Figure 2). Using this system, we were able to rapidly identify potential novel bacteriocin-producing LAB strains and discard those that produce known bacteriocins, thus accelerating the discovery of novel bacteriocins including variants of those already reported.

When the molecular mass of the bacteriocin is determined, supernatants with or without pretreatment of overnight culture with LAB strains are injected to the ESI-LC/MS system for analysis. In cases where bacteriocin production is low, in which direct LC/MS analysis of the supernatant is unable to visualize possible bacteriocin signals, sample pretreatment of the culture supernatants is essential to remove any media borne impurities, and to concentrate the bacteriocin in the supernatant. The mass detector is manually set at the mass range of *m/z *500-3000, which is the range of molecular mass for most bacteriocins [29]. This enabled us to determine the molecular mass of the active compound in the supernatant, which is compared to the molecular mass of reported bacteriocins. When molecular weights of the bacteriocins do not resemble any previously reported strains, we consider them novel. Moreover, in cases where no molecular masses are detected from the ESI-LC/MS analyses, we still classify them as potentially novel to minimize the possibility of false negative results.

For statistical analysis, antimicrobial spectra of the LAB strain are determined using the spot-on-lawn method [15] against a set of indicator strains (usually about 10 strains), and are analysed using PCA with the data sets from reported bacteriocins as the correlating variable. In most cases, bacteriocins are clustered into three groups, nisin variants, class IIa (anti-*Listeria*) bacteriocins, and narrow-spectrum bacteriocins. Based on our experience, most novel bacteriocins do not fall within any of these clusters. Nevertheless, novel bacteriocins falling within a distinct cluster are sometimes observed, especially novel variants of known bacteriocins. Thus, data obtained from the PCA should be combined with that from the molecular mass analysis [30].

## Structural determination and characterization of novel LAB bacteriocins

### Novel nisin variant

To illustrate the effectiveness of the screening system described above, one could refer to the paper that reported the discovery of the nisin variant, nisin Q [31]. Moreover, Zendo *et al*. (2008) clearly described the capacity of the system to discriminate between nisin variants A, Z, and Q [29]. Nisin Q, the third nisin variant to be reported, was isolated from *Lactococcus lactis *61-14, an LAB isolated from a Japanese river [31]. Although nisin Q appears to have similar structure to that of nisin A and Z, which were analysed by nuclear magnetic resonance (NMR) spectroscopy [32], gene annotation of the nisin Q locus revealed significantly lower similarity (only 82%) to other nisin variants, whereas nisin A and Z have almost identical gene loci. This indicates that, from an evolutionary perspective, the locus of nisin Q is at a greater genetic distance from the loci of both nisin A and Z. Nevertheless, nisin Q still shares the same biosynthetic pathway with other nisin variants as it was successfully produced when its structural gene, *nisQ*, was introduced to the nisin Z-producing strain together with nisin Z. Furthermore, nisin Q can stimulate pheromone activity in the regulation system of nisin A (NisRK), albeit to a much lower degree than that by nisin A [33].

**Figure 3:**
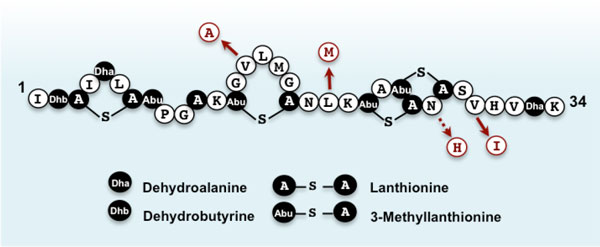
**Primary structures of nisin Q and its replacement amino acid residues relative to the other nisin variants, nisin A and Z**. Solid arrows indicate replacement residues for both nisin A and Z whereas broken arrow indicate nisin A. Unusual post-translationally modified amino acids such as dehydroalanine, dehydrobutyrine, lanthionine, and 3-methyllanthionine are indicated in black.

Nisin Q differs in four and three amino acid residues from nisin A and Z, respectively (Figure 3). While these nisin variants have comparable biochemical features and antimicrobial activity spectra, nisin Q is less susceptible to oxidation, a common phenomenon/reaction that significantly reduces the bioactivity of nisin, than the other derivatives [33]. The inherent high stability of nisin Q against oxidation is brought about by the absence of a methionine at the hinge region. Nisin A and Z have a methionine residue at position 21 in the central hinge region, whereas in nisin Q, methionine is replaced with leucine (Figure 3). Although nisin Q still has a methionine residue at position 17, this occurs in the rigid ring structure making it less susceptible to oxidization [34]. The increased oxidative resistance of nisin Q compared to the other variants suggests that this strain may be particularly useful in certain applications such as food systems where oxidation is common.

### Novel class IIa: anti-listerial bacteriocins

The high specificity of class IIa bacteriocins against the psychrophilic food pathogen *L. monocytogenes *has brought this group to prominence in its potential use against this lethal pathogen. Enterocin NKR-5-3C (Ent53C) is one of the bacteriocins produced by *Enterococcus faecium *NKR-5-3, an LAB isolated from the Thai fermented fish *Pla-ra *[35,36]. Ent53C showed very strong microbial activity (in nanomolar range) against *Listeria *spp. and other Gram-positive species, a typical characteristic of class IIa bacteriocins [35].

**Figure 4:**
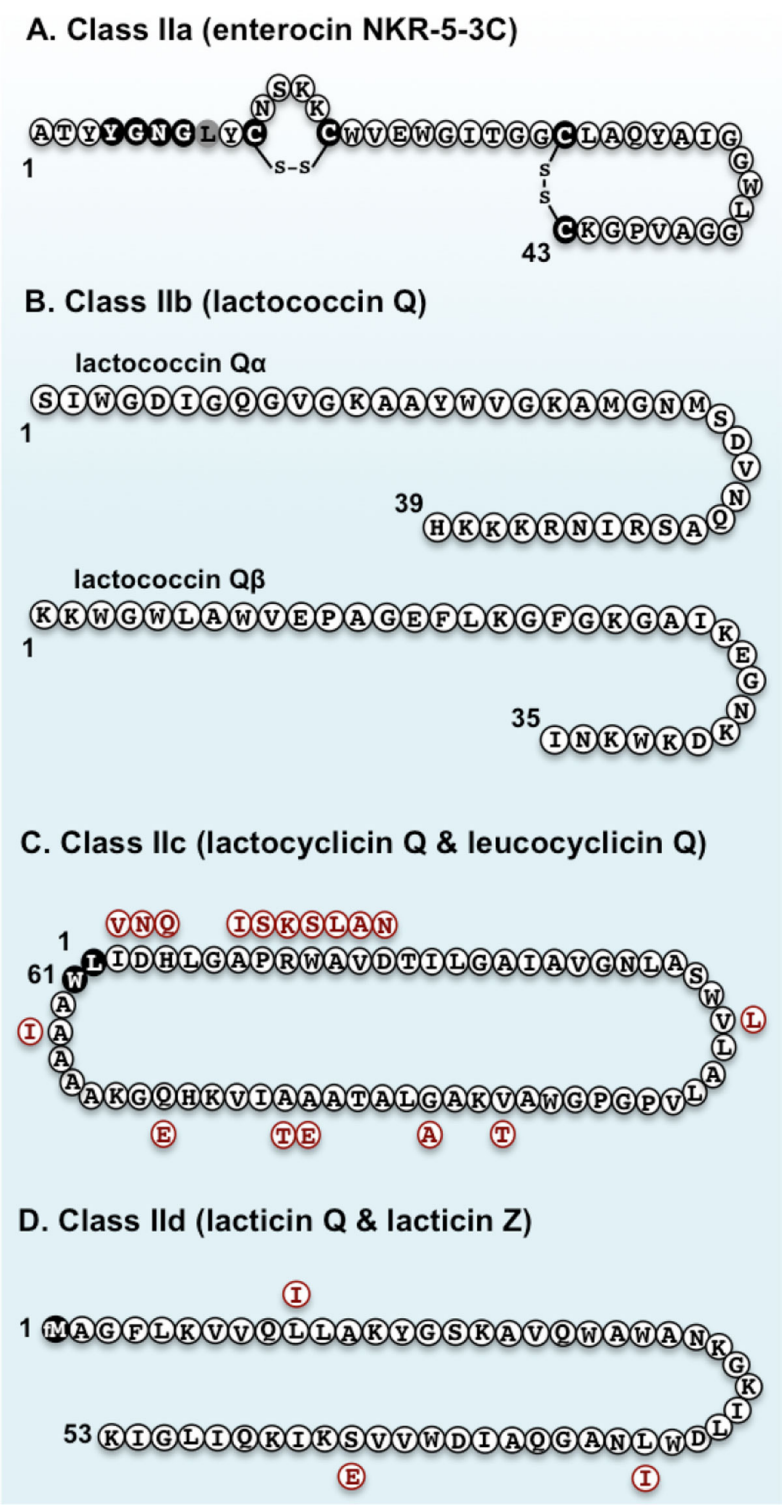
**Primary structures of representative class II bacteriocins isolated/studied in our laboratory**. Enterocin NKR-5-3C (Ent53C) is a novel class IIa (pediocin-like) bacteriocin. The conserved YGNGV (but in the case of Ent53C, V is replaced with L, shown in gray residue) and cysteine residues involved in disulfide bridges are highlighted in black (A). Lactococcin Q is a novel class IIb (two-peptide) bacteriocin, which is comprised of two peptides (Qα and Qβ) that show synergistic activity with each other (B). Lactocyclicin Q (LycQ) and leucocyclicin Q (LcyQ) are novel class IIc (circular) bacteriocins. Residues in red represent the replaced residues to constitute for LcyQ. N- and C- terminal residues involved in the head-to-tail cyclization are shown in dark residues (C). Lacticin Q and lacticin Z are leaderless (class IId) bacteriocins with a formylated methionine residue (fM) at the first N-terminal residue. Residues in red represent the replaced residues to constitute for lacticin Z (D).

Class IIa bacteriocins have a conserved sequence (YGNGV) known as the pediocin-box at their N-terminal region. In the pediocin-box of Ent53C, valine is replaced with leucine (Figure 4A). This is not uncommon, since a number of bacteriocins with a variant pediocin-box, YGNGL, have been reported [37-40]. However, among class IIa bacteriocins with a variant pediocin-box, only Ent53C contains two disulfide bridges [35]. The number of disulfide bridges in class IIa bacteriocins directly correlates with the intensity of their antimicrobial activity and stability [16,41]. The strong antimicrobial activity of Ent53C, especially against *Listeria *spp., and its exceptionally high stability as indicated by the number of disulfide bridges, should be useful for its future application in various fields.

### Novel class IIb: two-peptide bacteriocins

While most bacteriocins exist as a single active peptide molecule, two-peptide (class IIb) bacteriocins, as the name suggests, consist of two different individual peptide molecules that require equal amounts of each peptide to exert its optimal antimicrobial activity [17,42]. In our laboratory, we have three novel bacteriocins belonging to this class: lactococcin Q (Figure 4B) [43], enterocin X [44], and enterocin NKR-5-3AZ [36].

Lactococcin Q, a novel two-peptide bacteriocin isolated from *L. lactis *QU 4, an LAB isolated from corn, is comprised of two peptides: Qα and Qβ [43]. These two peptides show high similarity to lactococcin Gα and Gβ, peptides that comprise the two-peptide bacteriocin lactococcin G [45]. Both lactococcins G and Q have very narrow and specific antimicrobial spectra, with antimicrobial activity only against strains derived from *L. lactis *[43,45]. Structural analysis of lactococcin Q peptides showed the presence of α-helical structures in the same positions as in lactococcin G, thus suggesting that these bacteriocins have similar modes of action [43]. Furthermore, hybrid combinations of these homologous lactococcins have identical activity spectra to the original combinations, although some lead to activities distinct from the original. When mixed with an equimolar concentration of Gβ, Lactococcin Qα had 32 times lower specific activity than the original combination, whereas Gα and Qβ in combination had specific activity comparable to the original lactococcin Q combination [43].

Enterocin X is a novel two-peptide bacteriocin from *E. faecium *KU-B5 that has different activity against target microorganisms than to its component peptides, enterocins Xα and Xβ. When equimolar concentrations of these peptides were tested against a panel of indicator strains, the combined antimicrobial activity was not uniformly enhanced, with activities of 0.13-130-fold or 1.0-1020-fold than that of Xα and Xβ alone, respectively [44].

Conversely, enterocin NKR-5-3AZ (Ent53AZ) is one of the multiple bacteriocins produced by the bacterial strain *E. faecium *NKR-5-3 [36]. This bacteriocin showed very high homology to another two-peptide bacteriocin, brochocin-C, which is comprised of brochocins A and B [46]. Although purification of Ent53Z from the culture supernatant of strain NKR-5-3 was unsuccessful [36], as occurred with its homologue, brochocin B [46], its putative structural gene, *ent53Z*, was found directly downstream of the putative structural gene of Ent53A in the genome of NKR-5-3 [36].

### Novel class IIc: circular bacteriocins

Circular bacteriocins (class IIc) are characterized by their unique structural feature of a head-to-tail cyclization of their backbones [19,20]. The circular nature of their structure provides greater structural stability, higher thermal stress resistance, and superior stability against proteolytic digestion, compared to their linear counterparts [47,48]. Owing to their exceptional sturdiness, the use of circular bacteriocins as food preservatives and stable antimicrobial agents for clinical setting is seriously being considered. However, in order for this full potential to be recognized, it is important that their biosynthetic mechanisms are understood, which currently remains unknown.

In our laboratory, we have isolated two novel circular bacteriocins that share high identity with each other despite their producer strains belonging to distinct genera. Lactocyclicin Q (LycQ), isolated from *Lactococcus *sp. QU 12 [49], and leucocyclicin Q (LcyQ), isolated from *Leuconostoc mesenteroides *TK41401 [50], are both circular bacteriocins composed of 61 amino acid residues (Figure 4C). DNA sequence analysis of structural genes revealed their identical nature. LycQ and LcyQ share 72% DNA sequence identity. Moreover, both their precursor peptides contain a leader sequence of two amino acid residues in which cyclization (to yield the mature bacteriocin) occurs between L3 and W63 [50]. Secondary structure prediction analyses of both bacteriocins revealed four identical α-helices, all having subtle amphiphilic characteristics that are thought to play an important role in their antimicrobial action.

Like any typical circular bacteriocin, LycQ and LcyQ both exhibit high stability against thermal, pH, and proteolytic enzyme stresses [49,50]. For instance, LycQ maintains full activity after exposure to 121°C for 15 min under acidic condition, as well as exposure to a pH range of 3.0-9.0 [49]. Other bacteriocins, such as nisin Z, lose the majority of their antimicrobial activity when exposed to similar conditions [51].

As with other circular bacteriocins, the biosynthetic mechanisms of LycQ and LcyQ remain unclear. Although attempts to heterologously express LcyQ failed, identification of its putative biosynthetic gene cluster was recently reported by Mu *et al*. [52], who also identified the biological function of LcyD in the maturation of LcyQ. LcyD is a protein belonging to a large family of proteins of unknown functions - these are the DUF95 superfamily membrane proteins that are widespread in the biosynthetic gene clusters of circular bacteriocins [19,20].

### Novel leaderless bacteriocins (class IId)

While most bacteriocins are synthesized as inactive prepeptides, which contain an N-terminal leader peptide attached to the C-terminal propeptide moiety [10], some bacteriocins are atypical in the sense that they are synthesized without an N-terminal leader sequence. Since the leader peptides of typical bacteriocins have essential roles in recognition sites for secretion and maturation during the bacteriocin synthesis, as well as to protect the producer strain by keeping them in an inactive state while still inside the producer bacterial cells, leaderless bacteriocins are presumed to have unique biosynthetic mechanisms [10,53]. From an application perspective, leaderless bacteriocins have promising commercial potential since they can be readily synthesized without the need to cleave the leader peptide. This makes scale-up production considerably easier, even in heterologous eukaryotic production systems.

In our laboratory, we have isolated and studied a number of novel leaderless bacteriocins. For example, lacticin Q and its homologue lacticin Z were produced by *L. lactis *QU 5 and *L. lactis *QU 14 respectively. Strain QU 5 is an LAB isolated from corn [22] while strain QU 14 was isolated from horse intestine [54]. In addition, *Weissella hellenica *QU 13, an LAB isolated from Japanese pickles, *Takana-zuke*, was recently found to produce two novel leaderless bacteriocins, weissellicins Y and M [55].

Lacticin Q and Z share 94% identity, varying only in three amino acid residues at positions 10, 33, and 44. Furthermore, they both have a formylated-methionine at the initiation residue (Figure 4D). They are both 53-amino-acid highly cationic peptides that show very strong antimicrobial activity (at nanomolar concentrations) as well as high stability against various stresses [22,54]. Lacticin Q contains two amphiphilic helices that play an important role in its antimicrobial activity [56-58]. Owing to the very high homology and comparable activity spectra of these two leaderless bacteriocins, it was inferred that they have the same mode of antimicrobial action [54].

**Figure 5:**
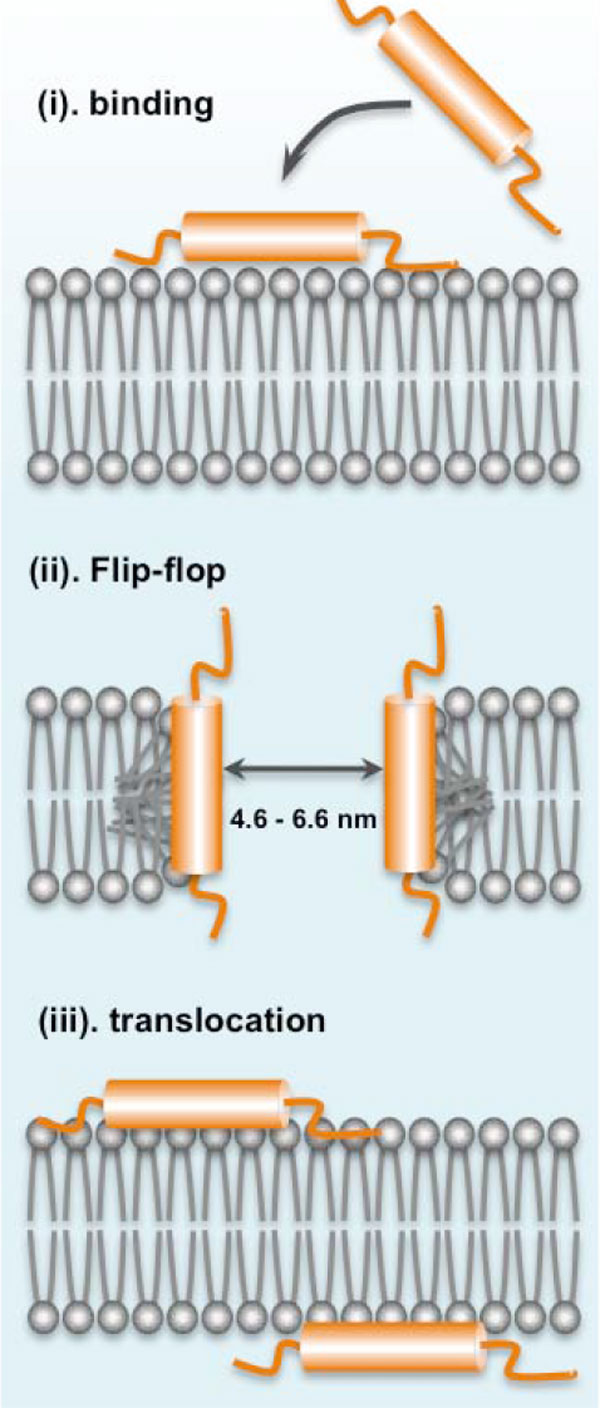
**Huge toroidal pore (HTP) model of the antimicrobial action of lacticin Q**. The highly cationic lacticin Q rapidly binds to the negatively charged phospholipid bilayer membrane (i) that would result in the formation of HTPs, coupled with membrane lipid flip-flop that would cause the leakage of intracellular components, including ions, ATP, and small proteins (ii), after which, the lacticin Q molecules translocate into the membrane as the pore closes (iii).

The antimicrobial action mechanism of lacticin Q was extensively studied. While most bacteriocins require a docking molecule for their antimicrobial action, lipid II for nisin A and other lantibiotics, and mannose ABC transporter, MptD, for pediocin PA-1/AcH and its homologue bacteriocins, lacticin Q was found to cause high-level membrane permeabilization of target strains without any specific receptors [57]. It was found that lacticin Q forms a huge toroidal pore (HTP) around 4.6-6.6 nm in size that is large enough to cause leakage of intracellular components such as ions and ATP as well as large molecules such as proteins resulting in cell death [56]. It was shown that the mechanism of HTP formation begins by the electrostatic interaction of the cationic lacticin Q molecules and the negatively charged membranes. The rapid binding of lacticin Q to the phospholipid bilayer membranes results in the formation of HTPs coupled with lipid flip-flop. Intracellular components then escape from these pores and cell death ensues. The pores formed in the membrane are short-lived because these HTPs close as the lacticin Q molecules translocate from the outer to the inner cell membrane (Figure 5) [56]. However, the killing mechanism through HTP formation of lacticin Q is selective and highly dependent on the physiological features of the outer membrane of target cells, which explains the non-toxicity of lacticin Q against Gram-negative bacteria [58]. Furthermore, it was recently suggested that another mechanism might be responsible for the selective antimicrobial activity of lacticin Q. Differences in the antimicrobial intensity of lacticin Q against some sensitive bacteria even within species may be due to accumulation of hydroxyl radicals through the Fenton reaction. It was inferred that the selective toxicity of lacticin Q would depend on the strains ability to scavenge these hydroxyl radicals [59].

The genes involved in the biosynthesis of lacticin Q and Z were recently elucidated [60]. The biosynthetic gene clusters *lnqBCDEF *and *lnzBCDEF *are involved in the secretion and self-immunity of lacticin Q and Z, respectively. Overexpression of *lnqQ *in *L. lactis *NZ9000 resulted in the intracellular accumulation of lacticin Q. This led to growth suppression as a result of the intracellular toxicity of the bacteriocin, but the growth suppression was circumvented when *lnqBCDEF *was co-expressed [60]. Further analyses on the functions of the gene products of *lnqBCDEF *revealed that secretion of lacticin Q to the extracellular space is strictly controlled by the gene products of this gene cluster while the self-immunity system is more flexible. The ABC transporter-type immunity LnqEF is sufficient to confer minimum immunity, whereas LnqBCD are thought to be accessory proteins that support the activity of LnqEF [61].

## Applications of LAB bacteriocins

LAB have been associated with food fermentations dating all the way back to ancient times due to their beneficial influence on nutrition, organoleptic, and shelf-life of foods [1]. However, it is their ability to produce antimicrobial peptides, bacteriocins, that have made them particularly promising in both food and pharmaceutical industries. In the food industry, bacteriocins have huge potential in the biopreservation of various foods, either alone, or in combination with other methods of preservation, which is known as hurdle technology [5]. The antimicrobial activity of many bacteriocins, especially the class IIa bacteriocins, against the highly pathogenic and food-borne *L. monocytogenes *offers an ideal solution to the problem caused by this sturdy pathogen, which is commonly reported to contaminate ready-to-eat refrigerated food products [5]. There are different approaches by which bacteriocins may be applied to food systems. While the use of bacteriocin as a food preservative in its purified form is the most commonly used approach, direct inoculation of bacteriocin-producing LAB into the food system is also effective. The pediocin PA-1/AcH-producing *Pediococcus pentosaceus *BCC 3772 when used as starter culture for *Nham*, a traditional Thai fermented pork sausage, effectively controlled the growth of *L. monocytogenes *without compromising the quality of *Nham *[62]. The utilization of the product that is fermented by a bacteriocin-producing LAB as a raw material for food processing has also been shown to be effective [63]. In addition, the incorporation of bacteriocins into the food packaging film or surfaces has also been explored [64,65]. In our recent study, when pediocin PA-1/AcH was incorporated into a biocomposite packaging film, the initial load of *L. monocytogenes *on the meat surface was significantly reduced [65].

The clinical potential of bacteriocins has been the subject of on-going investigation by many scientists all over the world due to the activity of some bacteriocins against Gram-positive human and animal pathogens, including MDR pathogens such as methicillin-resistant *Staphylococcus aureus *(MRSA) strain and vancomycin-resistant *Enterococcus faecalis *(VRE) strain [12,66,67]. In addition, biofilm associated infections among patients with indwelling medical devices have remained a challenge for medical researchers. In our recent study, we showed that nisin A and lacticin Q exert strong bactericidal activities against MRSA both in its planktonic and biofilm cells, whereas vancomycin, the commonly used antibiotic to treat MRSA infections, showed bactericidal activity against planktonic cells only [67].

Furthermore, because bacteriocins are ribosomally synthesized, they have relatively simpler biosynthetic mechanisms compared to those of conventional antibiotics. The gene-encoded nature of bacteriocins makes them easily amenable through bioengineering to either increase their activity or specify target microorganism. Owing to this feature of bacteriocins, antibiotic therapy would become less damaging to the natural gut microflora, which is a common drawback of conventional antibiotic use.

In Japan, as well as the utilization of nisin as a food preservative, we succeeded in developing a nisin A-containing hand wash and oral hygiene gel [68,69]. This is anchored on the food-grade distinction of nisin A, an important prerequisite of the government regulation for the application of bacteriocin in Japan [70]. This nisin A-containing hand wash is better in terms of stability and bioactivity compared to general hand wash. Although antimicrobial activities of most bacteriocins are limited against Gram-positive bacteria, it is well known that nisin and other bacteriocins become potent against Gram-negative pathogens when they are exposed to agents, such as surfactants, that could compromise the integrity of their outer membranes [71,72]. Whereas, Oralpeace™, was shown to be effective in controlling tooth cavities (caused by *Streptococcus mutans*) and bacterial gingivitis (caused by *Porphyromonas gingivalis*) [68,69,73]. Oralpeace™ contains only natural edible materials and compounds, making it a better option for individuals who have difficulty gargling, such as the elderly and physically challenged individuals. Furthermore, in order to prolong the effectiveness of nisin in application systems, we successfully developed a liposome-encapsulated nisin that showed significantly prolonged activity than "naked" nisin in inhibiting the synthesis of *S. mutans *glucan-biofilm [73].

On the other hand, the use of nisin in the veterinary industry as a preventive drug and as a cure for bovine mastitis has also been explored. Bovine mastitis is a disease that has a major economic impact, not only in Japan, but also in the worldwide dairy industry, as it is the major source of economic loss among cattle farmers. However, there is a rising concern regarding the rampant use of conventional antibiotics to control bovine mastitis, which has resulted in antibiotic contamination in milk. We have shown that our nisin-based injectable drug can control almost 99.9% of bacteria causing mastitis such as *Staphylococcus aureus *and *Streptococcus agalactiae *60 s after drug administration [74].

Furthermore, in order to gain insights and preempt any possible mechanism of the emergence of bacteriocin-resistant strains, we studied the two-component systems (TCSs) of various commensal strains to bacteriocin-producing LAB strains such as *S. aureus *and *S. mutans*. Two novel TCSs NsrRS (nisin A-resistant TCS) and LcrRS (lacticin 481-resistant TCS) in *S. mutans *were found to be associated with resistance against the lantibiotics nisin A, nukacin ISK-1, and lacticin 481. NsrRS regulates expression of the protein NsrX that functions like the nisin immunity protein NisI, which binds to the nisin molecule. LcrRS on the other hand, was found to regulate the expression of the ABC transporter LctFEG, which also functions as an immunity protein complex for lacticin 481 [75]. Additionally, in the case of *S. aureus*, we demonstrated that there are several TCS associated with its resistance to antimicrobial compounds produced by other bacteria. BraRS (previously reported to be associated with bacitracin resistance) system functions specifically to negate the antimicrobial action of nisin A and nukacin ISK-1, whereas GraRS (reportedly involved in susceptibility to cationic peptides such as defensins, gentamicin, and vancomycin) and VraRS (vancomycin resistance-associated TCS) confer broad-spectrum resistance against general cationic peptides and cell-wall synthesis inhibiting compounds, respectively [76].

## Conclusions

Bacteriocins from Generally Recognized as Safe (GRAS) LAB have continued to gain great interest among an increasing number of research groups due to their huge application potential both in food, and in pharmaceutical industries. In the food industry, bacteriocins have long been proposed as a solution to the problems of food spoilage and food-borne infections. However, up to now, nisin remains the only commercially available and industrially utilized bacteriocin despite a vast array of bacteriocins being discovered in the past two decades. It was suggested that the combined lack of awareness of what bacteriocins can achieve in food systems, and the lack of enthusiasm to move away from existing food-preservation techniques, is the reason for the under-utilization of bacteriocin in the food industry [12]. Fortunately, there is an upward trend of both consumer preference and regulatory demand toward minimally processed food products without the use of chemical preservatives. This provides a genuine opportunity that could jump-start the widespread application of bacteriocins in the food industry. However, the use of bioengineered bacteriocins for food applications could face consumer resistance as in the case Genetically Modified Organisms (GMO's). As anti-GMO advocates have always argued, safety of their use must first be thoroughly evaluated.

On the other hand, perhaps one of the greatest concerns that human kind is facing in the 21^st ^century is the growing problem of MDR pathogens and the rapidly decreasing antibiotic arsenal to combat them. Researchers around the world are scrambling for possible alternatives to address this problem. The high specific activity of some bacteriocins against clinical pathogens, even against MDR strains, offers a possible solution to this growing problem. But perhaps it is their amenability to bioengineering that makes them exceptionally promising. There is an increasing number of reported bioengineered bacteriocins with narrow spectrum of activity (which minimize damage to the natural gut flora), enhanced bioactivity, and higher stability. Furthermore, the cyclization mechanism of circular bacteriocins could open up new ways to engineer circular therapeutic compounds to enhance their stability.

The advancement of science and technology has led to an increase in bacteriocin research, in terms of both quality and quantity, and consequently more novel bacteriocins with unique properties have been reported. It is apparent that there is still more to learn about this family of peptide antibiotics. Thus, in order to further enhance the bacteriocin arsenal against these undesirable microorganisms (spoilage and pathogens) it is important, not only to advance the study (mode of antimicrobial action and their biosynthetic mechanisms) of known bacteriocins, but also to continue the search for more novel bacteriocins with promising properties.

## Competing interests

The authors declare that they have no competing interests.

## Authors' contributions

All authors defined the topic of the review. RHP drafted the manuscript. TZ and KS polished it. All authors read and approved the final manuscript.
